# PD-L1/PD-1 pathway: a potential neuroimmune target for pain relief

**DOI:** 10.1186/s13578-024-01227-3

**Published:** 2024-04-20

**Authors:** Daling Deng, Tianhao Zhang, Lulin Ma, Wenjing Zhao, Shiqian Huang, Kaixing Wang, Shaofang Shu, Xiangdong Chen

**Affiliations:** 1 Department of Anesthesiology, Union Hospital, Tongji Medical College, Huazhong University of Science and Technology, Wuhan, 430022 Hubei China; 2 Key Laboratory of Anesthesiology and Resuscitation, Huazhong University of Science and Technology, Ministry of Education, Wuhan, China; 3 Institute of Anesthesia and Critical Care Medicine, Union Hospital, Tongji Medical College, Huazhong University of Science and Technology, 430022 Wuhan, China

**Keywords:** PD-L1, PD-1, Pain, Neuroimmune, Neuroinflammation

## Abstract

Pain is a common symptom of many diseases with a high incidence rate. Clinically, drug treatment, as the main method to relieve pain at present, is often accompanied by different degrees of adverse reactions. Therefore, it is urgent to gain a profound understanding of the pain mechanisms in order to develop advantageous analgesic targets. The PD-L1/PD-1 pathway, an important inhibitory molecule in the immune system, has taken part in regulating neuroinflammation and immune response. Accumulating evidence indicates that the PD-L1/PD-1 pathway is aberrantly activated in various pain models. And blocking PD-L1/PD-1 pathway will aggravate pain behaviors. This review aims to summarize the emerging evidence on the role of the PD-L1/PD-1 pathway in alleviating pain and provide an overview of the mechanisms involved in pain resolution, including the regulation of macrophages, microglia, T cells, as well as nociceptor neurons. However, its underlying mechanism still needs to be further elucidated in the future. In conclusion, despite more deep researches are needed, these pioneering studies indicate that PD-L1/PD-1 may be a potential neuroimmune target for pain relief.

## Introduction

According to the International Association for the Study of Pain (IASP), pain is defined as an unpleasant sensory and emotional experience associated with, or resembling that associated with, actual or potential tissue damage [1, 2]. Pain can be categorized into two types, acute pain and chronic pain, based on the causes and duration of the pain. Acute pain can be controlled and mostly resolves within a week [3]. However, inadequacies in postoperative acute pain management can hinder recovery and even lead to the development of chronic pain [4]. Epidemiological survey shows that approximately 20% patients suffered transition from acute to chronic pain after surgery [5]. It has been estimated that the prevalence of pain, especially chronic pain, can be as high as 40% worldwide [6, 7]. Pain has gradually become one of the world’s public health problems [8]. The lack of effective pain treatment and management can be attributed to the unclear nature of pain mechanism. Therefore, it is urgent to profound understanding of the pain mechanisms in order to develop better treatment.

Programmed death ligand 1 (PD-L1) is a member of the B7 family and the first functional ligand of programmed death receptor 1 (PD-1) [9]. PD-1 is an important immune checkpoint receptor and with an essential immunoregulatory function [10, 11]. Various studies demonstrate that PD-L1/PD-1 can significantly impact the regulation of immune response and tolerance process [12, 13]. For instance, targeted potentiation of PD-1 shows potential for suppressing autoreactive T cells and alleviate autoimmune diseases [14]. And, exosomal PD-L1 from the tumor can suppress T cell activation in the draining lymph node and promote tumor growth in an immune-dependent fashion [15]. Recently, the critical role of PD-L1/PD-1 pathway in neuroimmune, neuroinflammation, and synaptic transmission and plasticity is gradually being recognized. These crucial features are significantly disrupted in chronic pain, with mounting evidence suggesting that targeting the PD-L1/PD-1 pathway may provide a promising approach to alleviate pain [16–18]. For instance, PD-L1/PD-1 pathway can effectively alleviate neuropathic pain and delay the process of nerve injury by inhibiting the excitability of nociceptive neurons and regulating neuroinflammation [16, 19]. Furthermore, PD-L1/PD-1 pathway can also inhibit the activation of microglia/macrophages and promote their polarization to M2 phenotype [20, 21]. Hence, controlling the PD-L1/PD-1 pathway’s effect on the immune system may be potentially alleviate pain.

This review focuses on the potential analgesic effect of PD-L1/PD-1, with the rationality of regulating the neuroimmune interaction. It aims to summarize the role of PD-L1/PD-1 pathway in pain from the perspective of neuroimmune. The analgesic effect of PD-L1/PD-1 pathway has significant implications for exploring pain pathogenesis and developing clinical treatments for pain relief.

## PD-L1/PD-1 overview

PD-1, as a member of the CD28 immunoglobulin superfamily and is a surface receptor protein with a single type I transmembrane domain [11, 22]. Its cytoplasmic domain harbors two phosphorylation sites, consisting of an immunoreceptor tyrosine-based inhibitory motif (ITIM) [22–24]. PD-1 is widely expressed on the surface of B cells [25], monocytes, and activated T cells [26]. Research has shown that PD-1 interacts with two ligands, PD-L1 (CD274) and PD-L2 (CD273) [27]. As PD-L2 expression is restricted to professional antigen-presenting cells (APCs) [22], research regarding the PD-L2/PD-1 signal pathway remains limited. PD-L1 expression can be detected in non-hematopoietic healthy tissue cells including endothelial cells and epithelial cells, and hematopoietic cells including lymphocytes, natural killer cells, dendritic cells (DCs), and macrophages [28–31]. Recent studies revealed that there is a certain level of PD-L1 and PD-1 expression in the neuroaxis of pain, including nerves, spinal cord [32], and dorsal root ganglion (DRG) [19, 33–37].

PD-L1/PD-1 pathway plays a pivotal role in various diseases. PD-L1/PD-1 have the capability to hinder T cell proliferation and function, reduce cytokines production, induce T cell depletion, and reduce motor ability [38–40]. In the tumor microenvironment, activating the immune system could enhance the effectiveness of anti-PD-1 or PD-L1 treatments [41, 42]. The up-regulation of PD-1 expression can play a negative regulation in the expression of IL-12 on monocytes/macrophages, thereby regulating the function of immune cells [43]. PD-L1/PD-1 signaling pathway in brain decreased the deposition of amyloid-β peptide (Aβ), suppressed neuroinflammation, and delayed the development of Alzheimer’s disease [44]. Furthermore, PD-L1/PD-1 pathway can also participate in regulating the pathophysiological processes of other diseases, such as colitis [45], brain injury [46], spinal cord injury, and acute and chronic pain [19], by regulating neuroimmune and neuroinflammation.

## The role of PD-L1/PD-1 pathway in pain

Mounting evidence suggest that PD-L1/PD-1 pathway of DRG, sciatic nerve, and spinal cord dorsal horn (SDH) plays an important role in various pain models, including acute pain [19, 47], inflammatory pain [34, 37], neuropathic pain [16–18, 48–50], and cancer pain [19, 35, 51].(Table 1) The increased expression of PD-L1/PD-1 pathway in trigeminal ganglia neurons and DRG neurons impedes migraine-like pain and formalin-induced acute inflammatory pain [19, 47]. In addition, electroacupuncture can exert analgesic effects by activating the PD-L1/PD-1 pathway [34, 52]. In the model of chronic constrictive injury of sciatic nerve (CCI) and spared nerve injury (SNI), studies have suggested deficiency of PD-L1 significantly increased pain hyperalgesia [18, 48, 49]. Similarly, the absence of PD-1 also aggravated mice’s hind-paw mechanical hypersensitivity [50]. Administration of exogenous PD-L1 apparently increases pain threshold in naïve mice and mice with bone cancer pain [19, 35]. Notably, the blockade of PD-1 with nivolumab induces allodynia during the early phases of bone cancer pain in mice [35]. In the same way, Wang et al. suggested that nivolumab initially increases pain thresholds but may offer long-term benefits in the attenuation of bone cancer pain [51]. This may be attributed to the transient pain-inducing effect of nivolumab. According to the report, the increase of maternal peripheral PD-L1 level and pain thresholds during pregnancy follow the same trend [53]. Late-pregnant mice exhibit efficient resistance to pain. Simultaneously, using the pregnancy mouse model, Tan et al. found that high PD-L1 levels in late-pregnant mice will decrease following delivery, which indicates that PD-L1 mediates pregnancy-induced analgesia [54]. In the treatment of non-small cell lung cancer (NSCLC) patients with morphine, abnormal increase of morphine 3-glucoside (M3G), as an active metabolite of morphine, upregulated the expression of PD-L1 and ultimately promoted tumor escape [55]. Moreover, Wang et al. demonstrated that morphine produces antinociception via the mu opioid receptor (MOR) and PD-1 is activated and participates in regulating the function of MOR in DRG neurons [32]. In PD-1−/− mice, antinociception caused by morphine was significantly reduced [32]. These studies indicate the potential involvement of the PD-L1/PD-1 pathway in mediating opioid analgesia.

**Table 1:** Overview of the expression and localization of PD-L1/PD-1 pathway in pain model

Disease Model		Expression	Cellular localization		Regulated cell subset	References
			PD-L1	PD-1		
Inflammatory pain	CFA-induced(mice)	spinal dorsal horn	neurons	neurons	/	[33]
		DRG, spinal nerve axons, spinal axon terminals	neurons	neurons	microglia	[32]
	CFA-induced(rat)	DRG	neurons	neurons	/	[34]
	Formalin- induced(mice)	DRG spinal cord	neurons	neurons	/	[19]
Neuropathic pain	CCI-induced(mice)	sciatic nerves	macrophages	/	T cells and macrophages	[18]
	Chemotherapeutic-induced (mice)	DRG, peripheral nervous tissue	macrophages	primary sensory neurons	/	[16]
		DRG spinal cord	neurons	neurons, microglia, astrocytes	macrophage, T-cell	[89]
	SCI-induced(mice)	spinal cord	astrocytes macrophages/microglia	macrophages/microglia	macrophages/microglia	[81]
		spinal cord	neurons, macrophages/microglia, astrocyte, endothelial cell, oligodendroglia	/	macrophages and microglia	[17]
		spinal cord	microglia	microglia	microglia	[90]
		spinal cord	macrophages, microglia, B cells, neutrophils, and γб T cells	Tregs	Tregs	[95]
	SNI-induced (mice)	tibial and peroneal nerve, DRG	/	/	macrophages and T cells	[49]
		DRG, spinal nerve axons, spinal axon terminals	neurons	neurons	Microglia	[32]
		spinal cord	microglia	/	microglia	[52]
	retrovirus infection-induced (mice)	lumbar spinal cord, DRG	/	/	CD4^+^ and CD8^+^T cells Microglia macrophages	[50]
postherpetic neuralgia	Varicella-zoster virus -induced(patients)	peripheral blood mononuclear cells	CD4^+^ T cells, CD8^+^ T cells	CD4^+^ T cells, CD8^+^ T cells	CD4^+^ T cells, CD8^+^ T cells	[56, 57]
Cancer pain	Bone cancer(mice)	tumor-bearing BM	tumor cells	macrophages and monocytes	/	[51]
		DRG	neurons	neurons	/	[35]
		DRG spinal cord	neurons	neurons	microglia	[32]
	Melanoma(mice)	DRG spinal cord	neurons	neurons	/	[19]
	Non-small cell lung cancer(mice)	cancer tissue	A549 and H1299 cells	Tumor cells	CTL, CD8^+^ T cells	[55]
	/(patients)	peripheral blood	T cells	T cells	T cells	[59]
others	Migraine-like pain(mice)	trigeminal ganglia	TG neurons	TG neurons	/	[47]
	Capsaicin-evoked (mice and patients)	DRG	neurons	neurons	/	[36]
	Pregnancy-Induced Pain(mice)	spinal cord	/	/	/	[54]

Note: CCI: chronic constrictive injury; SNI: spared nerve injury; CFA: complete Freund’s adjuvant; SCI: spinal cord injury; CTL: cytotoxic T lymphocytes.

In clinical, a study suggested that varicella-zoster virus (VZV) could productively modulate expression of immunoinhibitory proteins and blocking PD-L1 enhances virus specific CD8^+^ T cell effector function [56]. In VZV-induced postherpetic neuralgia (PHN), the expression of PD-L1 and PD-1 in T cells were higher in patients with PHN than without PHN [57]. Different exercise patterns alleviated the arthritic pain of patients with osteoarthritis, also accompanied by an increase in serum PD-1 levels [58]. Zhang et al. indicated that compared with normal people, the positive expression rate of sPD-1 and PD-1 are significantly higher in patients with cancer pain [59]. The expression of PD-1 on T cell surfaces decreased and peripheral sPD-1 content increased with increasing degree of cancer pain [59]. Similarly, Wang et al. also found that the overexpression of PD-L1 in NSCLC patients [60]. In addition, mounting studies have shown that PD-L1/PD-1 inhibitors may cause pain in cancer treatment [61]. For example, Majenka et al. have reported a series of acute low back pain due to administration of monoclonal antibodies directed against PD-1/PD-L1 for skin cancer treatment in patients [62]. And Melanoma and lung cancer patients may experience arthralgia following PD-1 inhibitor treatment [63–65].

In conclusion, PD-L1/PD-1 pathway is capable of inhibiting both physiological and pathological pain. Subsequently, we will elaborate on the mechanism of pain relief through the PD-L1/PD-1 pathway in detail.

## The mechanisms of PD-L1/PD-1 pathway in pain relief

### PD-L1/PD-1 pathway and macrophage

There is abundant evidence that macrophages are capable of accumulating at the nerve injury site and secrete various inflammatory mediators, thus sensitizing nociceptive neurons [66–69]. Macrophages, functioning as key regulators of peripheral pain, exert control over the inflammatory response and pain signaling through their interactions with neurons [70]. Thus, it is conceivable that macrophages, being pivotal immune cells, could be considered as potential targets for PD-L1/PD-1. The underlying mechanisms are as follows.

### PD-L1/PD-1 pathway inhibits macrophage proliferation and infiltration

A study has demonstrated that in the CCI model, PD-L1 expression on macrophages increased significantly, and the absence of PD-L1 leads to an upregulation of macrophage expression [18]. In SNI mice model, Karl et al. found that PD-L1 can induce infiltration of macrophages into the injured peroneal and tibial nerve. And compared with wild-type mice, PD-L1 knockout mice exhibited a higher increase in the number of macrophages in the peripheral injured nerves [49]. In addition, the up-regulation of PD-L1 induced by spinal cord injury (SCI) is accompanied by the accumulation of activated macrophages in peripheral organs [71]. Taken together, PD-L1/PD-1 can exert an analgesic effect by affecting the proliferation and infiltration of macrophages, although its precise mechanisms require further investigation.

### PD-L1/PD-1 pathway regulates macrophage polarization

Macrophage polarization stands out as a pivotal event contributing the progression of chronic pain [72–74]. Macrophages polarize into classically activated/inflammatory (M1) and alternatively activated/regenerative (M2) macrophages under certain conditions [75]. A number of pro-inflammatory cytokines (TNF- α, IL-6, CCL2/MCP1) released from M1 macrophages, have been shown to cause neuronal sensitization by stimulating their specific receptors [76]. On the other hand, M2 macrophages secrete anti-inflammatory cytokines (IL-10, TGF- β, and IL-4), which in turn suppresses pain responses [69, 77–80]. Yao et al. suggested that after SCI, the levels of PD-L1/PD-1 in macrophages of injured spinal cord were significantly increased. Similarly, in paclitaxel induced neuropathic pain, macrophages from the DRG were the main immune cells expressing PD-L1, and anti PD-L1 treatment increased the mechanical pain threshold and chronic neuropathy development by upregulating the expression of inflammatory factors TNF, IL-6 and Cx3cr1 in peripheral nerve tissue [16]. In PD-L1 knockout mice subjected to CCI, the sustained inflammatory response and severe mechanical hyperalgesia may be attributed to the diminished inhibition of the pro-inflammatory factors TNF- α and MCP-1 derived from macrophages [18, 48]. These suggested that PD-L1 inhibition may promote macrophage polarization towards M1 type, thereby increasing the secretion of pro-inflammatory mediators to trigger hyperalgesia. Deficiency of PD-1 promoted the polarization of macrophages to the M1 phenotype via enhancing the expression of p-STAT1 and downregulating the expression of p-STAT6 and augmented the proinflammatory cytokine TNF- α, IL-12, and IFN- γ secretion, thereby delaying the repair process of nerve injury after SCI [81]. Kong et al. also found that increased PD-L1 expression after SCI can inhibit the neuroinflammation response, promote motor function and sensory recovery, and alleviate neuropathic pain by inhibiting the MAPK signaling pathway and attenuating M1-like macrophage activation and promoting M2-like polarization [17]. Therefore, PD-L1/PD-1 has a vital role in the regulation of macrophage polarization. In future research, a comprehensive exploration of its regulatory mechanisms may pave the way for the development of innovative therapeutic approaches for managing pain.

### PD-L1/PD-1 pathway and microglia

Microglial cells are resident macrophages of the central nervous system, responsible for immune cells, clear cell debris, regulate synaptic plasticity, etc [82]. In the past decade, there has been more and more excellent research that focused on the role of microglia in pain [83–86]. Microglia are progressively gaining recognition as key regulators of various types of pain. Central pain sensitization is closely related to the activation of microglia [87, 88]. Increasing research has demonstrated that PD-L1/PD-1 pathway plays a regulatory role in microglia in pain and the potential mechanisms are elucidated below.

### PD-L1/PD-1 pathway inhibits microglia proliferation and activation

The study found that in the model of peripheral neuropathy caused by AIDS virus infection, deficiency of PD-1 accelerated the onset of mechanical allodynia and was associated with a significantly up-regulated number of microglia infiltrating the spinal cord and activation of resident microglia. Activated microglia elevated levels of iNOS and 3-nitrotyrosine in both small (IB4+) and large (NF200+) DRG sensory neurons and contribute to nerve damage and neuropathic pain [50]. It indicates that PD-L1/PD-1 reduces neuronal damage and alleviates peripheral neuropathy by mediating the activation of spinal microglia [50]. PD-1 has the capacity to inhibit the proliferation of microglia in SDH [32]. Similarly, Livni et al. showed that combined chemotherapy and anti-PD-1 treatment on peripheral neuropathy can induce the increase and activation of microglial cells in the dorsal horn of the spinal cord. Activated microglia contribute to the inhibition of sensory axon growth and the development of peripheral neuropathic pain [89]. In conclusion, PD-L1/PD-1 regulates neuroinflammation by inhibiting the proliferation and activation of microglia, thereby delaying the development of pain.

### PD-L1/PD-1 pathway regulates microglia polarization

The polarization of microglia is also essential for the development of pain. PD-L1/PD-1 pathway can regulate the state of microglia, promote the ratio of M1/M2 microglia reduced, improve motor dysfunction, and relieve pain after SCI [17, 81]. Deficiency of PD-1 will induce the polarization of microglia to the M1 phenotype via the activation of STAT1 and nuclear factor-kappa B and enhance the phagocytosis of microglia in the M1 and M2 phenotype, which is contrary to the regulation of PD-1 on phagocytosis of phagocytes [81]. The mechanism behind this difference still requires further elucidation. A recent study has shown that the up-regulation of PD-1 in the spinal dorsal horn of SCI rats drives the polarization of microglia to M2 phenotype by promoting AMPK signaling, participates in regulating the inhibition of neuroinflammation by dexmedetomidine, and accelerates the regeneration and repair of nerve tissue [90]. In addition, Wu et al. suggested that EA may promote the polarization of activated M1 microglia to M2 microglia through the PD-L1/PD-1 pathway inhibited the MAPK signaling pathway, to reduce inflammation and alleviate neuropathic pain induced by SNI [52]. In conclusion, as the main effector cells, microglia play an important role in the pathophysiological process of pain. PD-L1/PD-1 regulating microglia polarization is also a potential pathway to relieve pain.

### PD-L1/PD-1 pathway and T cells

T cells are derived from bone marrow lymphocytes and mainly participate in humoral immune response [91]. T cells also play a key role in pain development. Different T cell subsets can secrete different cytokines and play a “double-sword” role. For example, exogenous administration of CD8^+^T cells can aggravate neuropathic pain, while regulatory T cells (Tregs) significantly alleviate neuropathic pain [92]. There is plenty of evidence that PD-L1/PD-1 axis is a critical element in regulating T cell functions in different disease models [93, 94]. Consequently, T cells may represent another essential target to induce the analgesic effects of PD-L1/PD-1 pathway.

### PD-L1/PD-1 modulates the function of T cells

On the one hand, it was reported that the overexpression of PD-1 was implicated in the maintenance of the anti-inflammatory function carried out by Tregs infiltrating the spinal cord in the subacute phase of SCI. The knockout of PD-1 in Tregs decreased the production of IL-10, TGF-β, and Foxp3, and impaired the neuroprotective effects mediated by Tregs, resulting in the attenuation of the inhibitory activity of Tregs on pro-inflammatory macrophages/microglia [95]. Therefore, PD-1 plays an essential role in maintaining the inhibitory function of Tregs.

On the other hand, using the same mouse model, Diana M et al. showed that the upregulation of PD-L1/PD-1 was associated with the functional impairment of CD8^+^T cells, which could block the immune-inflammatory cascade and limit the spread of inflammation at the injured site [71]. Blocking PD-1 leads to an increase in the production of TNF-α by CD8^+^T cells, thereby restoring the proinflammatory function of CD8^+^ T cells and accelerating inflammatory response [96]. Besides, there is increasing evidence that PD-1 can be reversed the dysfunction of exhausted T cells in patients with neuropathic pain. The upregulation of PD-L1/PD-1 expression in nociceptive neurons can curtail the survival of CD8^+^T cells, and PD-L1/PD-1 impairs the immune response of CD8 + T cells [97, 98]. Likewise, Jones et al. suggested that blocking PD-L1 enhances virus-specific CD8^+^ T cell effector function, further substantiating the inhibitory role of the PD-L1/PD-1 pathway on the pro-inflammatory function of CD8^+^ T cells [56].

### PD-L1/PD-1 affects the proliferation of T cells

The study revealed a significant increase in the expression of PD-L1 on CD4^+^T cells and CD8^+^T cells in patients with postherpetic neuralgia (PHN), accompanied by a corresponding rise in the number of T cells [57]. Analogously, in sciatic nerves of PD-L1-deficient mice after CCI, there was an increase in T cell infiltration, which was associated with hyperalgesia in neuropathic pain [18]. Using enzyme-linked immunosorbent assay (ELISA) and flow cytometry, Zhang et al. found that the content of PD-L1 and PD-1 in peripheral blood of patients with cancer pain increased, and the ratio of PD-1^+^ T cells notably enhanced [59]. Furthermore, in tumor tissue of patients with non-small cell lung cancer (NSCLC) receiving opioid analgesia, M3G specifically bound to TLR4 and upregulated PD-L1 expression via the PI3K signaling pathway, the overexpression of PD-L1 negatively regulated the number and activation of cytotoxic T lymphocytes (CTL), which indicated that the upregulation of PD-L1/PD-1 affects the amount and function of human CTL, participating in the opioid analgesia mechanism of cancer pain patients [55].

All in all, in several pain models, PD-L1/PD-1 modulates the function and proliferation of T cells to alleviate pain. However, the precise mechanism by which PD-L1/PD-1 regulates T cells warrants further investigation. PD-L1/PD-1 mediated immune response of T cells may be expected to become one of a promising pathway for clinical treatment of pain.

### PD-L1/PD-1 pathway and nociceptor sensory neuron

Nociceptors, as specialized primary sensory neurons, play a pivotal role in orchestrating responses to noxious stimuli in the surrounding tissues, consequently mediating the sensation of pain [76]. The tissue innervated by nociceptor terminals highly expressed molecular sensors, including transient receptor potential channels (TRPs) and voltage-gated sodium channels (Nav) [68, 99]. Multiple studies have indicated that changes in excitability of neurons on nociceptors is critical for the development of pain [68]. PD-L1/PD-1 pathway has emerged as a potential mechanism for alleviating pain by regulating the excitability of nociceptive sensory neurons.

Liu et al. showed that PD-L1/PD-1 regulates MOR signaling and enhances the role of morphine in antinociception by suppressing calcium currents in DRG neurons, inhibiting excitatory synaptic transmission, and inducing outward currents in spinal cord neurons [32]. Exogenous administration of PD-L1 can induce analgesic effects by reducing the excitability of DRG nociceptive neurons. This effect is mediated through the PD-1/SHP pathway, resulting in the subsequent inhibition of sodium channels and activation of TREK2 K + channels [19]. In the bone cancer pain model, the upregulation of PD-L1 promotes the secretion of CCL2, which selectively activates C-fiber nociceptive neurons in DRG and drives the pathogenesis of bone cancer pain [51]. Furthermore, a study suggested that PD-L1 induced the phosphorylation of SHP-1 via PD-1 and dose-dependently suppressed TRPV1 currents in DRG neurons, participating in the inhibition of hyperalgesia [35]. Additionally, Meerschaert et al. also showed that PD-L1/PD-1 on nociceptive neurons can alleviate capsaicin-induced the spontaneous pain behavior by inhibiting TRPV1-mediated calcium signaling and blocking transmission of nociceptive receptors [36].

GABAergic signaling in the spinal dorsal horn is also critical element of pain relief. Emerging evidence indicated that PD-1 can regulate the GABAergic signal in neurons of spinal dorsal horn through SHP-1 activation, ERK phosphorylation and inhibit the excitability of neurons [33]. In wild-type mice, intrathecal injection of GABA receptor agonist could reverse CFA-induced inflammatory pain, but this phenomenon was not observed in PD-1 deficient mice [33]. This observation underscores the role of PD-L1/PD-1 in regulating the function of excitatory neurons and its involvement in the pain control process by modulating GABAergic signaling.

Collectively, PD-L1 can regulate the excitability of spinal dorsal horn or DRG neurons through PD-1, thus offering promise in the inhibition of inflammatory, neuropathic, and cancer-related pain. As a neuromodulator, PD-L1/PD-1 has a specific effect on nociceptive neurons, which may be of great significance for the development of new analgesic drugs in clinic.

## Conclusions

**Fig. 1:**
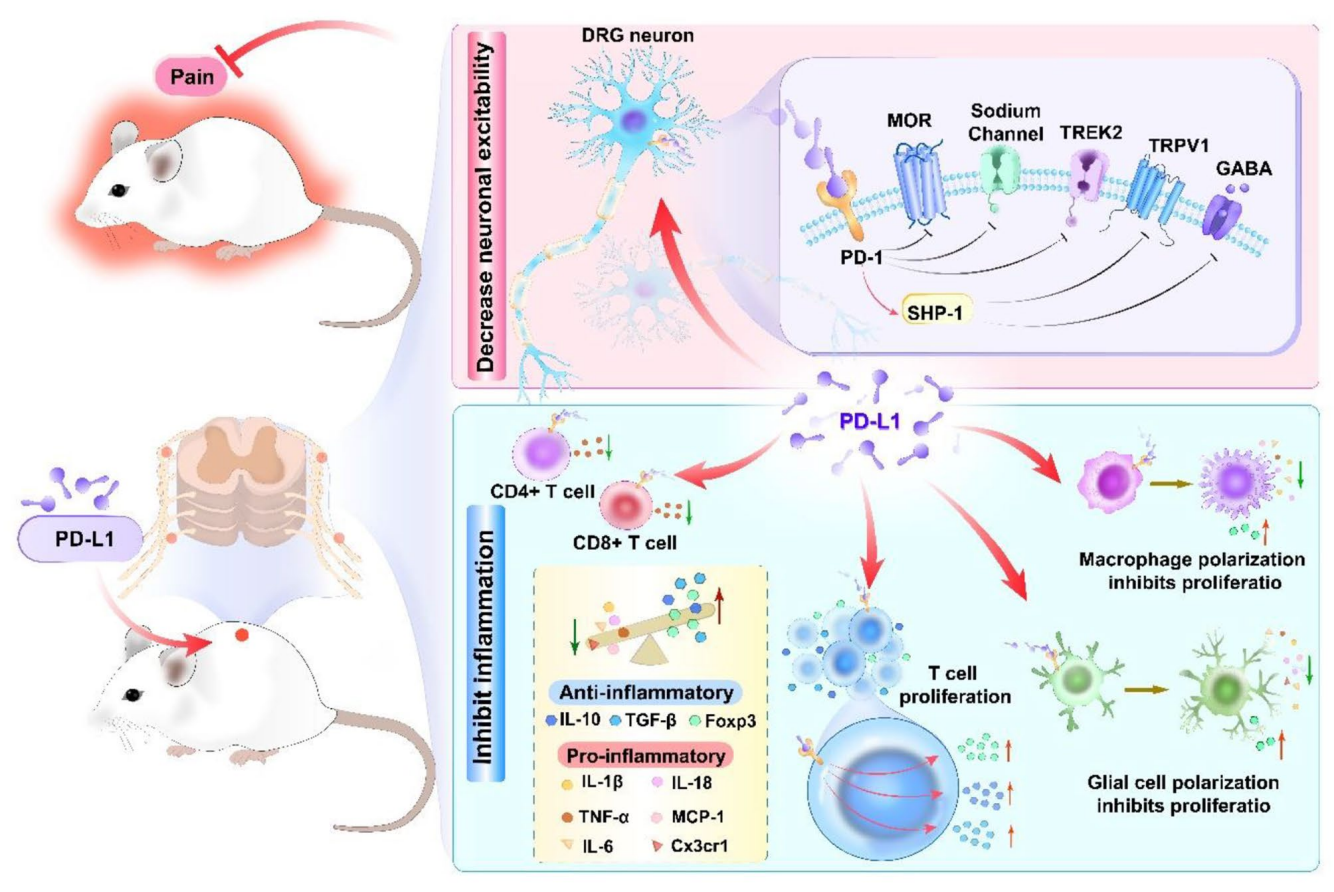
The mechanism overview of the PD-L1/PD-1 pathway in pain. The PD-L1/PD-1 pathway in pain relief through modulating macrophage/microglia cells, T cells, cytokines and neuronal

This review mainly from the perspective of PD-L1/PD-1 regulating immune cells and nociceptor neurons elaborates the mechanism of PD-L1/PD-1 alleviating pain. Additionally, it delves into the downstream signaling pathways implicated in the pathophysiological development of pathological pain (see Fig. 1). First of all, PD-L1/PD-1 activation subsequently regulates the proliferation and activation of macrophages, microglia, and T cells, promotes the polarization of macrophages/microglia, and ultimately alleviates the inflammatory responses and pain. Secondly, PD-L1/PD-1 activation contributes to the amelioration of pathological pain by downregulating the expression of pro-inflammatory cytokines and upregulating the expression of anti-inflammatory cytokines. In addition, PD-L1/PD-1 activation can also inhibit the ion channels of nociceptor sensory neurons and regulate the excitability of neurons. The efficacy of the PD-L1/PD-1 pathway in pain relief has been corroborated in diverse pain models, including the improvement effect of PD-L1/PD-1 activation on inflammatory pain, neuropathic pain, and cancer pain, indicating that activation of PD-L1/PD-1 may have broad applicability for treating pain. In a word, PD-L1/PD-1 is considered a potential analgesia target.

Besides, current studies indicate research on the role of PD-L1/PD-1 pathway in pain mainly focused on the peripheral nerve, DRG, and spinal cord levels. Nevertheless, the pathological processes involved in chronic pain also encompass various brain regions. PD-L1/PD-1 was also widely expressed in many brain regions, including hippocampal, cortical, hypothalamic, etc [31, 33, 100, 101]. PD-L1/PD-1 pathway has been gradually identified as being of primary importance to involve in various central nervous system diseases, such as stroke [46], tumors [102], and dementia [103]. Accumulating evidence suggested that PD-L1/PD-1 can regulate neuronal excitability, synaptic transmission, and plasticity, participating in processes such as learning, memory, anesthesia, and analgesia [30, 31, 104]. PD-L1/PD-1 may be as an important neuronal checkpoint. Despite these advancements, there is still a lack of research on the PD-L1/PD-1 pathway in brain regions involved in pain management, such as the anterior cingulate cortex and amygdala. Therefore, the role of the PD-L1/PD-1 pathway in different regions of the brain in chronic pain needs further exploration.

In clinical, PD-L1/PD-1 inhibitors function as immune checkpoint blockers, disrupting pathways associated with adaptive immune suppression [105], mainly targeting tumor immunotherapy. PD-L1/PD-1 checkpoint inhibitors are gradually becoming one of the main therapeutic agents for treating various cancer types including lung cancer [106, 107], gastrointestinal cancer [108, 109], melanoma [110, 111], among others. Studies indicate that a significant increase PD-L1 levels and PD-1 positivity in cancer patients, with a corresponding association with pain level [59, 60]. Due to a hyperactivated immune system [61], tumor anti-PD-L1/PD-1 immunotherapy can induce pain in cancer patients, including abdominal pain, arthralgia, acute low back pain, headache and so on [62–65, 112, 113]. In addition, research has shown that the use of opioids analgesics can have negative effects on cancer patients treated with PD-L1/PD-1 inhibitors [114, 115]. Over all, immunotherapy targeting the PD-L1/PD-1 pathway has a dual character, prescription opioids should be used with caution for tumor patients treated with PD-L1/PD-1 inhibitor and we should further study to optimize immunotherapy targeting the PD-L1/PD-1 pathway or develop combination therapies with PD-L1/PD-1 blockade to improve treatment efficiency and reduce side effects. In recent years, Zhao et al. demonstrated that small molecule analgesic peptide H-20, similar to PD-L1, can target PD-1 to alleviate acute and chronic pain with fewer side effects in several mouse models, which indicated that the development of analgesic drug can based on PD-L1/PD-1 axis as a candidate target [37]. However, further clinical research is needed to validate the role of PD-L1/PD-1 in pain.

In conclusion, PD-L1/PD-1 axis can be applied to pain relief as an important immune checkpoint. In future research, exploring the neuroimmune interaction of PD-L1/PD-1 pathway is conducive to the study of pain mechanism, providing a target for the follow-up treatment of pain, and developing new analgesic drugs.

## Data Availability

Data and materials are available upon request to corresponding author.

## Abbreviations

IASP: International Association for the Study of Pain
PD-L1: Programmed death ligand 1
PD-1: programmed death receptor 1
ITIM: Immunoreceptor tyrosine-based inhibitory motif
DCs: Dendritic cells
APCs: Antigen-presenting cells
SDH: spinal cord dorsal horn
DRG: dorsal root ganglion
NSCLC: non-small cell lung cancer
CCI: chronic constrictive injury
SNI: spared nerve injury
M3G: morphine 3-glucoside
MOR: mu opioid receptor
SCI: spinal cord injury
Tregs: regulatory T cells
CFA: complete Freund’s adjuvant
TRPs: transient receptor potential channels
Nav: voltage-gated sodium channels
CTL: cytotoxic T lymphocytes
